# Effectiveness of neoadjuvant immunochemotherapy compared to neoadjuvant chemotherapy in non-small cell lung cancer patients: Real-world data of a retrospective, dual-center study

**DOI:** 10.3389/fonc.2023.1145303

**Published:** 2023-03-30

**Authors:** Kailun Fei, Gang Guo, Jie Wang, Zhijie Wang, Yan Wang, Xuezhi Hao, Jia Zhong, Qinxiang Guo, Wei Guo, Wenzhong Su, Likun Zan, Jiaxi Xu, Fengwei Tan, Xiaofei Zhuang, Jianchun Duan

**Affiliations:** ^1^ State Key Laboratory of Molecular Oncology, National Cancer Center/National Clinical Research Center for Cancer/Cancer Hospital, Chinese Academy of Medical Sciences and Peking Union Medical College, Beijing, China; ^2^ Department of Medical Oncology, National Cancer Center/National Clinical Research Center for Cancer/Cancer Hospital, Chinese Academy of Medical Sciences and Peking Union Medical College, Beijing, China; ^3^ The Second Clinical Medical College of Shanxi Medical University, Taiyuan, China; ^4^ Department of Thoracic Surgery, Shanxi Province Cancer Hospital/Shanxi Hospital Affiliated to Cancer Hospital, Chinese Academy of Medical Sciences/Cancer Hospital Affiliated to Shanxi Medical University, Taiyuan, China; ^5^ Department of Medical Oncology, Shanxi Province Cancer Hospital/Shanxi Hospital Affiliated to Cancer Hospital, Chinese Academy of Medical Sciences/Cancer Hospital Affiliated to Shanxi Medical University, Taiyuan, China; ^6^ Department of Pathology, Shanxi Province Cancer Hospital/Shanxi Hospital Affiliated to Cancer Hospital, Chinese Academy of Medical Sciences/Cancer Hospital Affiliated to Shanxi Medical University, Taiyuan, China; ^7^ Department of Thoracic Surgery, National Cancer Center/National Clinical Research Center for Cancer/Cancer Hospital, Chinese Academy of Medical Sciences and Peking Union Medical College, Beijing, China; ^8^ Department of Cardiothoracic Surgery, Lvliang People’s Hospital, Lvliang, Shanxi, China

**Keywords:** non-small cell lung cancer, neoadjuvant immunochemotherapy, neoadjuvant chemotherapy, pathological response, real world study

## Abstract

**Background:**

Studying the application of neoadjuvant immunochemotherapy (NICT) in the real world and evaluating its effectiveness and safety in comparison with neoadjuvant chemotherapy (NCT) are critically important.

**Methods:**

This study included the II-IIIB stage non-small cell lung cancer (NSCLC) patients receiving NCT with or without PD-1 inhibitors and undergoing surgery after neoadjuvant treatments between January 2019 to August 2022. The clinical characteristics and treatment outcomes were retrospectively reviewed and analyzed.

**Results:**

A total of 66 patients receiving NICT and 101 patients receiving NCT were included in this study. As compared to NCT, NICT showed similar safety while not increasing the surgical difficulty. The ORR in the NICT and NCT groups was 74.2% and 53.5%, respectively, *P* = 0.009. A total of 44 patients (66.7%) in the NICT group and 21 patients (20.8%) in the NCT group showed major pathology response (MPR) (*P <*0.001). The pathology complete response (pCR) rate was also significantly higher in NICT group than that in NCT group (45.5% vs. 10.9%, *P <*0.001). After Propensity Score Matching (PSM), 42 pairs of patients were included in the analysis. The results showed no significant difference in the ORR between the two groups (52.3% vs. 43.2%, *P* = 0.118), and the proportions of MPR (76.2%) and pCR (50.0%) in NICT group were significantly higher than those of MPR (11.9%) and pCR (4.7%) in the NCT group (*P <*0.001). The patients with driver mutations might also benefit from NICT.

**Conclusions:**

As compared to NCT, the NICT could significantly increase the proportions of patients with pCR and MPR without increasing the operation-related bleeding and operation time.

## Introduction

Non-small cell lung cancer (NSCLC) is a highly invasive cancer type. Some patients show recurrence even after surgery. Among the locally advanced NSCLC patients, a recurrence rate of 70% and a long-term survival rate of less than 30% are observed even after radical surgery (1, 2). The five-year event-free survival (EFS) rate of NSCLC patients ranges from 68% for those with stage IB to 36% for those with stage IIIA (2). By inducing the downstaging of a tumor, preoperative chemotherapy might increase the R0 resection rate for patients with stage IB-IIIA NSCLC. Although preoperative chemotherapy has shown marginal improvements, the survival rate is only 5.4% higher than that of surgery alone (3). For neoadjuvant chemotherapy (NCT), only a few patients have shown pathological complete response (pCR) (median: 4%, range: 0 to 16%) and major pathological response (MPR) (4–8). Numerous studies have confirmed that pCR and MPR are closely correlated with local control, disease free survival (DFS), and overall survival (OS). Moreover, pCR and MPR are used as potential early predictors of survival (9).

The anti-programmed cell death 1 (PD-1)/programmed cell death ligand 1 (PD-L1) immunotherapies have revolutionized the treatment of metastatic and advanced-stage NSCLC (10, 11). The NADIM and LCMC-3 studies demonstrated the potential of neoadjuvant immunotherapy for NSCLC patients (12, 13). The CHECKMATE-816 study, a phase III clinical trial, confirmed that as compared to chemotherapy, neoadjuvant immunochemotherapy (NICT) could significantly increase the number of patients with pCR (24.0% vs. 2.2%) among the patients with stage IB-IIIA NSCLC, while the combination of neoadjuvant immunotherapy with chemotherapy remarkably prolonged the EFS (31.6 months vs. 20.8 months) (14). The NADIM II study highlighted that as compared to NCT, the NICT improved the MPR rate of patients with stage IIIA-B NSCLC (52.6% vs. 13.8%), enabling more patients to receive surgical treatment (93% vs. 69%) (15). However, whether the application of neoadjuvant therapy will increase the difficulty of surgery is an important concern. The previous studies suggested that neoadjuvant immunotherapy could slightly increase drug-related adverse reactions but did not significantly increase the risk of surgery.

These studies supported the application of immunochemotherapy for neoadjuvant treatment. However, the patients in clinical settings are highly selected, and the efficacy of NICT in the real-world environment requires further investigation. Based on this, the current study retrospectively analyzed the real-world data of NICT in Cancer Hospital, Chinese Academy of Medical Sciences and Shanxi Provincial Cancer Hospital to explore its effectiveness and safety.

## Patients and methods

### Patients

In this study, the resectable NSCLC patients treated with NCT with or without PD-1 inhibitors at two centers, including the National Cancer Center/National Clinical Research Center for Cancer/Cancer Hospital, Chinese Academy of Medical Sciences and Peking Union Medical College, Beijing, and Shanxi Provincial Cancer Hospital of Chinese Academy of Medical Sciences, Shanxi, from January 2019 to August 2022. The inclusion criteria were as follows: 1) the patients with stage II-IIIB NSCLC confirmed using imaging and histological examination before surgery; 2) the patients, who received feasible neoadjuvant therapy after an assessment; 3) the patients with Eastern Cooperative Oncology Group (ECOG) performance status (PS) score of 0 or 1; and 4) the patients, who underwent surgery after neoadjuvant treatment. Two researchers reviewed the clinical data of the patients from both centers, screened the neoadjuvant patients, which met the requirements, and collected the clinical baseline information, treatment response, and follow-up data. The baseline data included age, gender, and smoking history, and clinical features included comorbidities, primary lesion size, and location, while efficacy evaluation included imaging evaluation, pathological evaluation, and adverse reactions.

### Treatment methods

A total of 167 patients were divided into the NICT group (n = 66, treated with neoadjuvant PD-1 inhibitors in combination with chemotherapy) and the NCT group (n = 101, treated with chemotherapy only). All patients received conventional platinum-based doublets chemotherapy (cisplatin/carboplatin/nedaplatin/lobaplatin) (21 days per cycle). For the NICT group patients, Tislelizumab (26 cases, 39.4%), Camrelizumab (12 cases, 18.2%), Nivoliumab (10 cases, 15.2%), Pembrolizumab (9 cases, 13.6%), Sintilizumab (8 cases, 12.1%), and Toripalimab (1 case, 1.5%) were used. All the patients received video-assisted thoracic surgery (VATS) or traditional open thoracotomy.

### Evaluation

The image evaluation was performed by the researchers based on Response Evaluation Criteria in Solid Tumors version 1.1 (RECIST v1.1). Imaging was performed every two cycles or before the operation, and the treatment effects were evaluated by comparing preoperative images with baseline images. Objective response rate (ORR) was defined as the proportion of patients, who have a complete response (CR) or partial response (PR) to the treatments; it was evaluated based on RECIST v1.1. The pathological assessment was performed after surgery by professional pathologists based on the proportion of remaining tumor cells. If the proportion of residual tumor cells was less than 10%, it was defined as an MPR, and if no tumor cells were remaining, it was defined as pCR (16, 17). The treatment-related adverse events were assessed based on the Common Terminology Criteria for Adverse Events (CTCAE) version 5.0 published by the US Department of Health and Human Services (18).

### Follow up

During follow-up, the data of time to recurrence, site of recurrence, and survival date were collected. The patients were followed up once every three months for two year and every six months till November, 2022 (endpoint of study). At the endpoint of the study, the patients, who had not yet relapsed and those, who were still alive were analyzed for disease-free and overall survival. DFS was defined as the time from the patient’s surgery until the first discovery of disease recurrence or death, and OS was defined as the time from the discovery of the disease to the last follow-up or death of the patient.

### Statistical analyses

The continuous variables were expressed as means, medians, standard deviations, and ranges and analyzed using the Mann-Whitney U test and Kruskal-Wallis test to measure the best response outcome. The categorical variables were expressed as frequency and relative frequency and analyzed using Fisher’s exact test. The DFS of patients was identified using the Kaplan–Meier (KM) survival curve analysis and log-rank test. The clinical characteristics, including age, gender, smoking habits, comorbidity, ECOG score, cT stage, cN stage, and cTNM, histology, differentiation, and neoadjuvant treatment choice, were balanced using the PSM method in both the groups and analyzed using the nearest-neighbor method with a ratio of 1:1 without replacement and a 0.02-caliper width.

## Results

### Baseline characteristics of the patients

A total of 167 patients with resectable NSCLC, including 66 patients receiving NICT and 101 patients receiving NCT, were enrolled in this study. There were no significant differences in the clinical characteristics and baseline demographic characteristics between the two groups (**Table 1**). The majority of the enrolled patients were males, accounting for 89.4% and 80.2% of the NICT and NCT groups, respectively (*P* = 0.136), while the patients with ages above 60 years accounted for 40.9% and 50.5% in the two groups, respectively (*P* = 0.268). There were no significant differences in smoking and drinking habits and comorbidities of the patients between the two groups. Most patients showed excellent PS before neoadjuvant treatment in both groups (ECOG = 0, 45.5% and 1, 54.5%). The cT staging, cN staging, and cTNM grading before neoadjuvant therapy were similar between the two groups, showing no significant difference. Lung squamous cell carcinoma was the main pathological type among the NSCLC patients in both groups (78.8% and 64.4%, *P* = 0.057). Additionally, there were cases of squamous cell carcinoma with neuroendocrine differentiation in the NICT (4 cases, 6.1%) and NCT (2 cases, 2.0%) groups. Moreover, there was also one patient with adeno-squamous carcinoma in the NICT group. The pathological differentiation degree of patients mainly included low differentiation, accounting for 62.1% and 72.3% in the NICT and NCT groups, respectively (*P* = 0.374). Imaging showed that the tumor maximum tumor diameter (MTDs) in both groups were 5.383 cm and 4.500 cm respectively (*P* = 0.049). Both the groups received 1-4 cycles of neoadjuvant therapy before surgery, and most patients (75.8% and 76.2% in the NICT and NCT groups, respectively) underwent radical surgical resection of NSCLC after two cycles of neoadjuvant therapy. In this study, the number of patients receiving cisplatin chemotherapy was significantly more in the NCT group (51.5%) as compared to those in the NICT group (15.2%, *P <*0.001).

**Table 1:** Baseline clinical and sociodemographic characteristics of patients.

	Total n=167 (%)	Type of neoadjuvant treatment		*p*-value
		NICT n=66 (%)	NCT n=101(%)	
Sex				
Male, n (%)	140 (83.8%)	59 (89.4%)	81 (80.2%)	0.136
Female, n (%)	27 (16.2%)	7 (10.6%)	20 (19.8%)	
Age				
≥60 years, n (%)	78 (46.7%)	27 (40.9%)	51 (50.5%)	0.268
<60 years, n (%)	89 (53.3%)	39 (59.1%)	50 (49.5%)	
Smoking history				
Yes, n (%)	122 (73.1%)	51 (77.3%)	71 (70.3%)	0.374
No, n (%)	45 (26.9%)	15 (22.7%)	30 (29.7%)	
Drinking history				
Yes, n (%)	60 (35.9%)	25 (37.9%)	35 (34.7%)	0.742
No, n (%)	107 (64.1%)	41 (62.1%)	66 (65.3%)	
Comorbidity				
Yes, n (%)	61 (18,3%)	25 (18.9%)	36 (17.8%)	0.885
No, n (%)	273 (81.7%)	107 (81.1%)	166 (82.2%)	
ECOG				
0, n (%)	76 (45.5%)	30 (45.5%)	46 (45.5%)	1.000
1, n (%)	91 (54,5%)	36 (54,5%)	55 (54,5%)	
MTD in imaging (cm)		5.383 ± 1.871	4.500 ± 1.997	0.049
Clinical T stage				
cTl, n (%)	18 (10.1%)	6 (7.8%)	12 (11.9%)	0.509
cT2, n (%)	60 (33.7%)	23 (29.9%)	37 (36.6%)	
cT3, n (%)	56 (31.5%)	26 (33.8%)	30 (29.7%)	
cT4, n (%)	44 (24.7%)	22 (28.6%)	22 (21.8%)	
Clinical N stage				
N0,n (%)	14 (8.4%)	8 (12.1%)	6 (5.9%)	0.194
N1,n (%)	49 (29.3%)	22 (33.3%)	27 (26.7%)	
N2,n (%)	104 (62.3%)	36 (54.5%)	68 (67.3%)	
Clinical TNM stage				
IIA, n (%)	2 (1.2%)	1 (1.5%)	1 (1.0%)	0.253
IIB, n (%)	21 (12.6%)	12 (18.2%)	9 (8.9%)	
IIIA, n (%)	102 (61.1%)	36 (54.5%)	66 (65.3%)	
IIIB, n (%)	42 (25.1%)	17 (25.8%)	25 (24.8%)	
Histology type				
Squamous	117 (70.1%)	52 (78.8%)	65 (64.4%)	0.057
Non-squamous	50 (29.9%)	14 (21.2%)	36 (35.6%)	
Pathological differentiation				
High	2 (1.2%)	1 (1.5%)	1 (1.0%)	0.374
Medium	51 (30.5%)	24 (36.4%)	27 (26.7%)	
Low	114 (68.3%)	41 (62.1%)	73 (72.3%)	
Treatment cycles				
1 cycles	11 (6.6%)	3 (4.5%)	8 (7.9%)	0.807
2 cycles	127 (76.0%)	50 (75.8%)	77 (76.2%)	
3 cycles	22 (13.2%)	10 (15.2%)	12 (11.9%)	
4 cycles	7 (4.2%)	3 (4.5%)	4 (4.0%)	
Median (IQR)		2 ( ± 0)	2 ( ± 0)	
Cisplatin				
Yes, n (%)	62 (37.1%)	10 (15.2%)	52 (51.5%)	<0.001
No, n (%)	105 (62.9%)	56 (84.8%)	49 (48.5%)	

NCT, neoadjuvant chemotherapy; NICT, neoadjuvant immunochemotherapy; MTD, maximum tumor diameter; IQR, interquartile range.

PSM analysis was used to more accurately evaluate and compared the effectiveness and safety of NICT and NCT. PSM analysis included twelve baseline factors, including age, gender, smoking habits, comorbidity, ECOG score, cT stage, cN stage, cTNM, histology, differentiation, cycles of neoadjuvant treatment, and balance of cisplatin usage between the two groups (**Table 2**). A total of 42 patients receiving NICT and 42 patients receiving NCT were matched.

**Table 2:** Baseline clinical and sociodemographic characteristics of patients after PSM.

	Total n=84 (%)	Type of neoadjuvant treatment		*p*-value
		NICT n=42 (%)	NCT n=42 (%)	
Sex				
Male, n (%)	73 (86.9%)	37 (88.1%)	36 (85.6%)	0.748
Female, n (%)	11 (13.1%)	5 (11.9%)	6 (14.4%)	
Age				
≥60 years, n (%)	43 (51.2%)	23 (54.8%)	20 (47.6%)	0.513
<60 years, n (%)	41 (48.8%)	19 (45.2%)	22 (52.4%)	
Smoking history				
Yes, n (%)	59 (70.2%)	32 (76.2%)	27 (64.3%)	0.233
No, n (%)	25 (29.8%)	10 (23.8%)	15 (35.7%)	
Comorbidity				
Yes, n (%)	34 (40.5%)	18 (42.9%)	16 (38.1%)	0.657
No, n (%)	50 (59.5%)	24 (57.1%)	26 (61.9%)	
ECOG				
0, n (%)	13 (15.5%)	6 (14.3%)	7 (16.7%)	0.763
1, n (%)	71 (84.5%)	36 (85.7%)	35 (83.3%)	
Clinical T stage				
cTl, n (%)	11 (13.1%)	4 (9.6%)	7 (16.7%)	0.363
cT2, n (%)	33 (39.3%)	14 (33.3%)	19 (45.2%)	
cT3, n (%)	23 (27.4%)	14 (33.3%)	9 (21.4%)	
cT4, n (%)	17 (20.2%)	10 (28.6%)	7 (16.7%)	
Clinical N stage				
N0,n (%)	8 (9.5%)	5 (11.9%)	3 (3.6%)	0.696
N1,n (%)	27 (32.1%)	14 (33.3%)	13 (31.0%)	
N2,n (%)	49 (58.3%)	23 (54.8%)	26 (61.9%)	
Clinical TNM stage				
IIA, n (%)	3 (3.6%)	1 (2.4%)	2 (4.8%)	0.544
IIB, n (%)	12 (14.3%)	6 (14.3%)	6 (14.3%)	
IIIA, n (%)	59 (70.2%)	28 (66.7%)	31 (73.8%)	
IIIB, n (%)	10 (11.9%)	7 (16.7%)	3 (7.1%)	
Pathological type				
Squamous	60 (70.1%)	30 (78.8%)	30 (64.4%)	1.000
Non-squamous	24 (29.9%)	12 (21.2%)	12 (35.6%)	
Pathological differentiation				
Medium	40 (47.6%)	24 (57.1%)	16 (38.1%)	0.081
Low	44 (52.4%)	18 (42.9%)	26 (61.9%)	
Treatment cycles				
1 cycles	7 (8.3%)	2 (4.8%)	5 (11.9%)	0.689
2 cycles	63 (75.0%)	33 (78.6%)	30 (71.4%)	
3 cycles	8 (9.5%)	4 (9.5%)	4 (9.5%)	
4 cycles	6 (7.1%)	3 (7.1%)	3 (7.1%)	
Median (IQR)		2 ( ± 0)	2 ( ± 0)	
Cisplatin				
Yes, n (%)	40 (47.6%)	21 (50.0%)	19 (45.2%)	0.662
No, n (%)	44 (52.4%)	21 (50.0%)	23 (54.8%)	

NCT, neoadjuvant chemotherapy; NICT, neoadjuvant immunochemotherapy; IQR, interquartile range.

### Perioperative-related indicators

All the patients underwent radical tumor surgery within 16 to 42 days of the last cycle of neoadjuvant therapy with no surgical delay (with intervals exceeding the prescribed 42 days). Most patients in the NICT and NCT groups, accounting for 81.8% and 67.3% (*P* = 0.050), respectively, were mainly assisted by VATS. It could not be determined that the NICT group patients were more conducive to using VATS surgery (**Table 3**). Unilateral lobectomy was the main choice for the patients in the NICT and NCT groups (56.1% and 49.5%, respectively). Extensive resection was mainly unilateral lobectomy or unilateral combined lobectomy (18.2% or 13.6% in the NICT group and 15.8% or 23.8% in the NCT group, respectively). Wedge resection and sleeve resection were used for 1.5% and 10.6% of the NICT group patients and 1.0% and 9.9% of the NCT group patients, respectively. There were no significant differences in tumor resection (*P* = 0.581) and operation time (150 min and 170 min, respectively, for the NICT and NCT groups) (*P* = 0.108). The amount of blood loss from the patients in the NICT group (50.0 ± 227.2 mL) was significantly lower than that in the NCT group (170.000 ± 142.663 mL, *P <*0.001). There were no perioperative-related deaths or re-hospitalization due to surgical complications in both groups.

**Table 3:** Comparison of treatment modality and surgical outcomes for NSCLC patients.

	Total n=167 (%)	Type of neoadjuvant treatment		*p*-value
		NICT n=66 (%)	NCT n=101(%)	
Extent of resection				
Pneumonectomy	28(16.8%)	12(18.2%)	16(15.8%)	0.581
Lobectomy	87(52.1%)	37(56.1%)	50(49.5%)	
Bilobectomy	33(19.8%)	9(13.6%)	24(23.8%)	
Local resection	2(1.2%)	1(1.5%)	1(1.0%)	
Sleeve	17(10.2%)	7(10.6%)	10(9.9%)	
Operation time (min)		150.000 ± 50.439	170.000 ± 59.186	0.108
Intraoperative blood loss (ml)		50.000 ± 227.182	170.000 ± 142.663	<0.001
Total lymph nodes resected		19.32 ± 9.46	16.31 ± 5.43	0.198

VATS, video-assisted thoracic surgery; Mann-Whitney U test and Fisher’s exact test were used.

The post-PSM analysis (**Table 4**) showed that the incidence of drug-related adverse reactions in both groups was slightly 47.6%, and none of them were above grade 3 in both groups. There were no significant differences between the two groups in terms of surgical approach, resection range, operation time, and the number of lymph node dissections. The blood loss from the patients in the NICT group was still lower than that from the patients in the NCT group (176.13 ± 264.93 mL vs. 182.42 ± 162.37 mL, *P* = 0.025).

**Table 4:** Comparison of treatment modality and surgical outcomes for NSCLC patients after PSM.

	Total n=84 (%)	Type of neoadjuvant treatment		*p*-value
		NICT n=42 (%)	NCT n=42(%)	
TRAEs related to drugs				
Yes	40 (47.6%)	20 (47.6%)	20 (47.6%)	1.000
No	44 (52.4%)	22 (52.4%)	22 (52.4%)	
Surgical approach				
Thoracotomy	23 (27.4%)	8(19.0%)	15 (35.7%)	0.087
VATS	61 (72.6%)	34 (81.0%)	27 (64.3%)	
Extent of resection				
Pneumonectomy	11 (13.1%)	4 (9.5%)	7 (16.7%)	0.632
Lobectomy	41 (48.8%)	22 (52.4%)	19 (45.2%)	
Bilobectomy	24 (28.6%)	11 (26.2%)	13 (31.0%)	
Sleeve	8 (9.5%)	5 (11.9%)	3 (7.1%)	
Operation time (min)		166.22 ± 50.55	169.10 ± 52.11	0.763
Intraoperative blood loss (ml)		176.13 ± 264.93	182.42 ± 162.37	0.025
R0 resection				
Yes, n(%)	80 (95.2%)	41 (15.2%)	39 (51.5%)	0.608
No, n (%)	4 (4.8%)	1 (84.8%)	3 (48.5%)	
Total lymph nodes resected		18.54 ± 10.46	15.31 ± 6.43	0.208

VATS, video-assisted thoracic surgery; Mann-Whitney U test and Fisher’s exact test were used.

### pCR and MPR

As listed in **Table 5**, radiographic response evaluation was performed in all the patients before surgery, showing the ORR of 74.2% and 53.5% in the NICT and NCT groups, respectively (*P* = 0.009). The pathological analysis after surgery showed significantly higher MPR in 44 patients (66.7%) in the NICT group as compared to that in 21 patients (20.8%) in the NCT group (*P <*0.001). The pCR rate was also significantly higher in the NICT group than that in the NCT group. (45.5% vs. 10.9%, *P <*0.001). After PSM, there was no significant difference in ORR between the NICT and NCT groups (52.3% vs. 43.2%, *P* = 0.118), and the proportions of MPR (76.2%) and pCR (50.0%) were significantly higher in the NICT group than those (11.9% and 4.7%, respectively) in the NCT group (*P <*0.001).

**Table 5:** Comparison of treatment effectiveness in NSCLC patients.

	Before PSM		p-value	After PSM		*p*-value
	NICT n=66 (%)	NCT n=101(%)		NICT n=44 (%)	NCT n=44(%)	
ORR(PR+CR)						
Yes, n (%)	49 (74.2%)	54 (53.5%)	0.009	22 (52.3%)	19 (43.2%)	0.118
No, n (%)	17 (25.8%)	47 (46.5%)		20 (47.7%)	23 (56.8%)	
pCR						
Yes, n(%)	30 (45.5%)	11 (10.9%)	<0.001	21 (50.0%)	2 (4.7%)	<0.001
No, n (%)	36 (54.5%)	90 (89.1%)		21 (50.0%)	40 (95.3%)	
MPR						
Yes, n(%)	44 (66.7%)	21 (20.8%)	<0.001	32 (76.2%)	5 (11.9%)	<0.001
No, n (%)	22 (33.3%)	80 (79.2%)		10 (23.8%)	37 (88.1%)	

ORR, Objective response rate, the proportion of patients who typically achieved a 30% reduction in tumor volume and maintained it for more than 4 weeks; total response (CR) and partial response (PR).

### Outcomes of DFS

**Figure 1:**
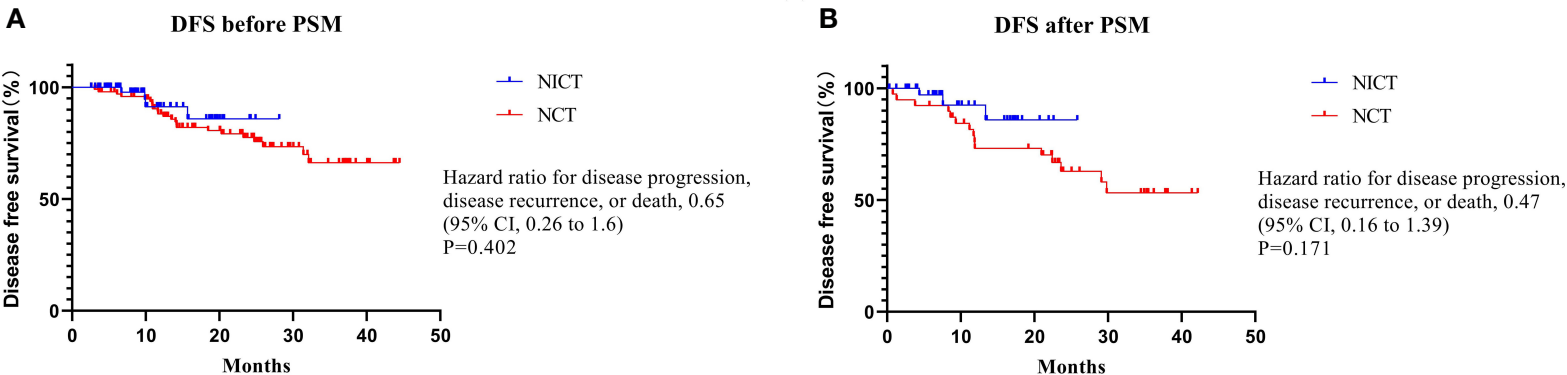
Disease free survival Summary. There was no statistically significant difference in DFS before or after PSM.

At the endpoint of this study (November 15, 2022), the median follow-up of patients in the NICT group was 9.7 months (range: 2.5-28.7 months), while that of patients in the NCT group was 23.0 months (range: 2.6-44.5 months). Only 4 (6.1%) and 23 (22.8%) patients in NICT and NCT groups, respectively, showed disease recurrence, which might be due to the limited follow-up time. After PSM, as compared to the NCT group, the NICT group showed a decreasing trend in the disease progression risk; however, the difference was not statistically significant (HR 0.46, 95% CI, 0.17-1.25, *P* = 0.171) (**Figure 1**).

### Efficacy in patients with driver mutations

In the NICT and NCT groups, a total of 19 and 39 patients underwent genetic screening after surgery, respectively. The results identified 5 and 13 patients with driver mutations in the NICT and NCT groups, respectively. The specific mutation information of the patients is listed in **Supplementary Table S1**. Due to the limited number of cases, statistical analysis was not conducted. In the NICT group, 3 (60%) patients with positive driver mutation showed MPR (one patient showed pCR), and 3 (23.1%) patients showed MPR (one patient showed pCR) in the NCT group (**Table 6**).

**Table 6:** Pathological response of patients with driver mutations and wild-type.

	Driver mutation		Wild-type	
	NICT n=5 (%)	NCT n=13 (%)	NICT n=61 (%)	NCT n=88 (%)
pCR				
Yes, n (%)	1 (20.0%)	1 (7.7%)	29 (47.5%)	10 (11.4%)
No, n (%)	4 (80.0%)	12 (92.3%)	32 (52.5%)	78 (88.6%)
MPR				
Yes, n (%)	3 (60.0%)	3 (23.1%)	41 (67.2%)	18 (20.5%)
No, n (%)	2 (40.0%)	10 (76.9%)	20 (32.8%)	67 (79.5%)

Due to the limited numbers of cases, statistical analysis was not conducted.

## Discussion

In this study, the efficacy and safety of NICT and NCT were compared using dual-center real-world data. The results showed that NICT resulted in a significantly higher pCR ratio (45.5% vs. 10.9%, *P <*0.001) and MPR ratio (66.7% vs. 20.8%, *P <*0.001) as compared to those of the NCT group, which might reduce the risk of disease recurrence. Furthermore, the baseline and neoadjuvant treatment characteristics of the two groups were balanced using the PSM method, which verified that NICT significantly improved the pCR and MPR without increasing the surgical risk.

The CHECKMATE-816 study confirmed that the application of NICT in NSCLC patients could improve the EFS of patients as compared with that of NCT. However, the real-world data comparing NICT with NCT is still relatively limited (19, 20). Therefore, the current study compared the data of dual-center NICT with NCT. The results suggested that NICT might improve the pCR and MPR of patients.

Several phase III studies and meta-analyses suggested that as compared to surgery alone, NCT could reduce the death risk of NSCLC patients by 13% to 16% and show a 5% benefit in their five-year survival (3, 7, 21). A PSM study analyzed 92 pairs of patients with cT2-4N0-1M0 NSCLC receiving adjuvant chemotherapy or NCT. The results showed no significant difference in the prognosis of patients between the two groups. Compared with surgery alone, neoadjuvant chemotherapy and adjuvant chemotherapy can improve the 5-year survival rate by about 5% (22). NCT could significantly improve the prognosis of patients as compared to surgery alone, but about 5% of the patients receiving NCT could not accept surgery due to disease progression, adverse reactions, and other factors (6, 7); therefore, NCT is mostly used for the patients, who are initially at risk of failing to achieve R0 resection. Compared with NCT, NCT combined with radiotherapy can further improve the R0 resection rate and prognosis of patients, but neoadjuvant radiotherapy improves the incidence of postoperative complications, so it is not widely used. The CHECKMATE-816 study included patients with stage IB to IIIA [according to 7^th^ edition American Joint Committee on Cancer (AJCC)] NSCLC. The subgroup analysis revealed that stage IIIA or IB-II NSCLC patients treated with NICT could obtain higher pCR and MPR rates. However, the NICT could significantly improve the EFS of patients with stage IIIA disease only. For patients with IB-II NSCLC, whether the improvement of pCR and MPR rates can translate into EFS and OS benefits remains to be further studied. For patients with stage IIIA-B, whether NICT can replace neoadjuvant radiotherapy and chemotherapy remains to be further explored. For stage IIIA-B patients, NICT can improve MPR rate and EFS, and has the potential to replace NCT combined with radiotherapy.

The 7th edition of the AJCC staging manual classified T3N2M0 patients as stage IIIA patients, while the 8^th^ edition classified them as stage IIIB patients (2). The patients with stage IIIB NSCLC in this study were initially resectable patients, excluding patients with N3 lymph node-positive. For the N2-positive patients, due to the risk of failing to achieve R0 resection, other treatment options, including concurrent chemoradiotherapy, surgery after neoadjuvant chemoradiotherapy, and surgery after induced chemotherapy are available (23–25). For operatable patients, surgery can improve the OS. As compared to NCT, NICT can improve the prognosis; however, it might cause difficulty in surgery, thereby limiting its application. Based on this study and previous studies, as compared with NCT, NICT did not lead to longer operation time, more bleeding, and higher perioperative complication rates (19, 26). Therefore, NICT might become one of the best treatment options for N2-positive patients.

Unlike the previous studies, which did not include patients with driver mutations or did not involve relevant gene screening, this study included some patients with driver mutations, the details of which are provided in **Supplementary Table 1**. In this study, the NICT group included 3 patients with epidermal growth factor receptor (EGFR) mutations, one patient with anaplastic lymphoma tyrosine kinase gene (ALK) fusion, and one patient with ROS proto-oncogene 1 (ROS1) fusion. Among these, 3 patients showed MPR (including one pCR). The NCT group included 9 patients with EGFR mutations, 3 patients with ALK fusion, and one patient with ROS1 fusion. Among these, only 3 patients showed MPR (including one pCR). This proportion was significantly lower than that in the NICT treatment group. All patients with EGFR mutation received EGFR-TKIs adjuvant treatment after surgery, while patients with ALK and ROS1 fusion did not receive targeted drug treatment after surgery, and none of the above patients had disease recurrence. Previous studies suggested that the MPR rate of patients with EGFR mutations, receiving neoadjuvant targeted therapy, was low (5% to 24.2%) (27–30). A multicenter study suggested that the patients with positive driver genes might still benefit from NICT treatment (31); these results were consistent with those observed in the our study. The selection of perioperative treatment and improvement the survival of these patients by NICT require further exploration.

As a retrospective study, this study was limited by the sample size and included only 42 patients in the analysis after PSM. This limitation made the subgroup analysis of patients, benefiting from NICT, impossible. Due to the short follow-up time and the difference of median follow-up time between the two groups, the data on DFS and OS in this study were not mature yet; therefore, the validity of NICT was evaluated using pCR and MPR as potential alternative endpoints. Therefore, for many problems, including the best applicable population of NICT and the best perioperative treatment strategy for patients with driver gene mutations, further prospective randomized controlled study is needed to explore.

In conclusion, using the dual-center real-world data, this study suggested that in clinical practices, the selection of patients with new adjuvant therapy could be based on the resectable patients with II-IIIB stage NSCLC. As compared with NCT, NICT could significantly increase the proportion of pCR and MPR in the patients without increasing the operation-related bleeding and operation time.

## Data availability statement

The raw data supporting the conclusions of this article will be made available by the authors, without undue reservation.

## Ethics statement

The studies involving human participants were reviewed and approved by Chinese Academy of Medical Sciences and Peking Union Medical College. Written informed consent for participation was not required for this study in accordance with the national legislation and the institutional requirements.

## Author contributions

Conceptualization, JD and XZ; validation, JD and FT; data collection, KF, GG, ZW YW, JZ, XH, WG, and WS; statistical analysis, KF and GG; writing original draft preparation, KF and GG; writing-editing, JD and JX; supervision, YW, JW, ZW, QG, and LZ. All authors contributed to the article and approved the submitted version.
