# Supplementary material for: Effectiveness of neoadjuvant immunochemotherapy compared to neoadjuvant chemotherapy in non-small cell lung cancer patients: Real-world data of a retrospective, dual-center study
Source: Front Oncol. 2023 Mar 30;13:1145303. doi: 10.3389/fonc.2023.1145303 (PMC10098217; doi:10.3389/fonc.2023.1145303)
Supplement: Supplementary file 1 [file Table_1.docx]

Supplementary Table S1. Details of patients with driver mutation.

|  | Mutation | Stage | Treatment | Pathology response |
| --- | --- | --- | --- | --- |
| 1 | EGFR L858R | IIIA | NCT | Non-MPR |
| 2 | EGFR L858R | IIIA | NCT | Non-MPR |
| 3 | ALK-EML4 | IIIA | NCT | Non-MPR |
| 4 | EGFR L858R | IIIA | NCT | MPR |
| 5 | ALK-EML4 | IIIB | NCT | Non-MPR |
| 6 | ALK-EML4 | IIIA | NCT | Non-MPR |
| 7 | ROS1 fusion | IIIA | NCT | Non-MPR |
| 8 | EGFR 19del | IIIA | NCT | MPR |
| 9 | EGFR 19del | IIIA | NCT | Non-MPR |
| 10 | EGFR 19del | IIIB | NCT | pCR |
| 11 | EGFR 19del | IIIA | NCT | Non-MPR |
| 12 | EGFR 19del | IIIA | NCT | Non-MPR |
| 13 | EGFR L858R | IIIA | NCT | Non-MPR |
| 14 | EGFR L858R | IIIA | NICT | Non-MPR |
| 15 | EGFR L858R | IIIA | NICT | MPR |
| 16 | ROS1 fusion | IIB | NICT | pCR |
| 17 | EGFR 19del | IIIA | NICT | MPR |
| 18 | ALK-EML4 | IIIA | NICT | Non-MPR |
